# Reducing the use of empiric antibiotic therapy in COVID-19 on hospital admission

**DOI:** 10.1186/s12879-021-06219-z

**Published:** 2021-06-02

**Authors:** Natasha N. Pettit, Cynthia T. Nguyen, Alison K. Lew, Palak H. Bhagat, Allison Nelson, Gregory Olson, Jessica P. Ridgway, Mai T. Pho, Jade Pagkas-Bather

**Affiliations:** 1 Department of Pharmacy, The University of Chicago Medicine, 5841 S Maryland Ave, Chicago, IL 60637 USA; 2 Department of Medicine, Section of Infectious Diseases and Global Health, The University of Chicago Medicine, Chicago, IL USA

**Keywords:** Antimicrobial stewardship, COVID-19, Pneumonia

## Abstract

**Background:**

Empiric antibiotics for community acquired bacterial pneumonia (CABP) are often prescribed to patients with COVID-19, despite a low reported incidence of co-infections. Stewardship interventions targeted at facilitating appropriate antibiotic prescribing for CABP among COVID-19 patients are needed. We developed a guideline for antibiotic initiation and discontinuation for CABP in COVID-19 patients. The purpose of this study was to assess the impact of this intervention on the duration of empiric CABP antibiotic therapy among patients with COVID-19.

**Methods:**

This was a single-center, retrospective, quasi-experimental study of adult patients admitted between 3/1/2020 to 4/25/2020 with COVID-19 pneumonia, who were initiated on empiric CABP antibiotics. Patients were excluded if they were initiated on antibiotics > 48 h following admission or if another source of infection was identified. The primary outcome was the duration of antibiotic therapy (DOT) prior to the guideline (March 1 to March27, 2020) and after guideline implementation (March 28 to April 25, 2020). We also evaluated the clinical outcomes (mortality, readmissions, length of stay) among those initiated on empiric CABP antibiotics.

**Results:**

A total of 506 patients with COVID-19 were evaluated, 102 pre-intervention and 404 post-intervention. Prior to the intervention, 74.5% (*n* = 76) of patients with COVID-19 received empiric antibiotics compared to only 42% of patients post-intervention (*n* = 170), *p* < 0.001. The median DOT in the post-intervention group was 1.3 days shorter (p < 0.001) than the pre-intervention group, and antibiotics directed at atypical bacteria DOT was reduced by 2.8 days (*p* < 0.001). More patients in the post-intervention group were initiated on antibiotics based on criteria consistent with our guideline (68% versus 87%, *p* = 0.001). There were no differences between groups in terms of clinical outcomes.

**Conclusion:**

Following the implementation of a guideline outlining recommendations for initiating and discontinuing antibiotics for CABP among COVID-19 inpatients, we observed a reduction in antibiotic prescribing and DOT. The guideline also resulted in a significant increase in the rate of guideline-congruent empiric antibiotic initiation.

**Supplementary Information:**

The online version contains supplementary material available at 10.1186/s12879-021-06219-z.

## Background

Patients admitted to the hospital with coronavirus disease 2019 (COVID-19) are often prescribed empiric antibiotic therapy for possible community-acquired bacterial pneumonia (CABP), as presenting symptoms are difficult to distinguish between viral or bacterial etiologies. However, the widespread prescribing of empiric antibiotics for possible bacterial pneumonia is not well supported by available literature regarding co-infections in the setting of COVID-19 [1–5]. A recent review identified that despite a low incidence (8%) of reported co-infections among patients with COVID-19, 72% of patients receive antimicrobial therapy [1]. While initiating empiric antibiotics for CABP may be reasonable, antibiotic therapy should be re-evaluated once COVID-19 pneumonia is confirmed. Prescribing empiric antibiotics when the clinical presentation is inconsistent with bacterial pneumonia or continuing antibiotics longer than necessary should be avoided in order to minimize the potential for adverse consequences.

It is well established that antibiotic use results in increased rates of antimicrobial resistance and increased risk of antibiotic-associated complications such as *Clostridioides difficile* infection and antibiotic related toxicities [6]. Antimicrobial stewardship interventions may facilitate avoidance of unnecessary antibiotic prescribing among patients with COVID-19 and help front-line clinicians make decisions regarding appropriate initiation and de-escalation of antibiotics for CABP based on laboratory data and chest imaging. In an effort to reduce unnecessary prescribing of empiric antibiotics among COVID-19 inpatients at The University of Chicago, the Antimicrobial Stewardship Program (ASP) and Infectious Diseases COVID-19 Consult Service developed guidance for antibiotic initiation and discontinuation. The purpose of this study was to determine the impact of this intervention on the prescribing of antibiotics for CABP among COVID-19 patients.

## Methods

This single-center, quasi-experimental, retrospective cohort study was conducted at an 811-bed academic medical center in Chicago, IL, USA. All adult patients admitted with COVID-19, confirmed by SARS-CoV-2 testing (nasopharyngeal swab), between March 1, 2020 and April 25, 2020 who received at least one dose of empiric antibiotics for CABP initiated within 48 h of admission were included. Patients were excluded if another source of infection was identified that was not pneumonia for which antibiotics were indicated and initiated. According to University of Chicago Medicine institutional policy, this project underwent a formal administrative review and was determined to be Quality Improvement. As such, this initiative was deemed not human subjects research and was therefore not reviewed by the Institutional Review Board. All data was de-identified when reviewed for this analysis.

On March 27, 2020, the Antimicrobial Stewardship Program (ASP) in conjunction with ID providers outlined recommendations regarding when to initiate antibiotics for possible bacterial pneumonia and when to discontinue empiric antibiotics among patients with COVID-19 (Supplementary Material, Figure 1). These recommendations were incorporated into the institution’s inpatient COVID-19 management guideline. Indications to initiate empiric antibiotics for CABP included the presence of leukocytosis, fever, or chest imaging suggestive of a bacterial process. The guideline also included recommendations for ordering a respiratory bacteria and viral panel (RBVP; Biofire Diagnostics FilmArray® respiratory Panel, Biomerieux, Salt Lake City, UT), a *Legionella* urinary antigen, and a *Streptococcus pneumoniae* urinary antigen. Discontinuation of atypical coverage (e.g. azithromycin, doxycycline, or levofloxacin) was recommended in patients with a negative *Legionella* urinary antigen and a RBVP negative for atypical bacterial pathogens. Additionally, discontinuation of other  antibiotics prescribed for CABP (e.g. ceftriaxone or cefdinir) was recommended in patients with negative RBVPs for non-atypical bacterial pathogens and a negative *Streptococcus pneumoniae* urinary antigen.

Throughout the study period, recommendations and education regarding antibiotic use among COVID-19 inpatients were given to COVID-19 unit providers during daily virtual rounds with the ID COVID-19 Consult Service [7]. Education was also provided to emergency department (ED) staff. All admitted patients with confirmed COVID-19 received an automatic ID consult for evaluation of antibiotic therapy in addition to COVID-specific management. Each ID consult team included an ID/ASP pharmacist who, along with the ID providers, evaluated each patient case. After updating the institution’s guideline to include recommendations for CABP, this evaluation also included a standardized and targeted stewardship intervention to recommend obtaining an RVBP, *Streptococcus pneumoniae* urinary antigen and/or *Legionella* urinary antigen (if not performed on admission), along with recommendations to discontinue or de-escalate antibiotics for CABP, in accordance with the institutional guideline.

The primary endpoint was the median duration of antibiotic therapy for CABP between two time periods during the COVID-19 pandemic, March 1 to March 27 (pre-intervention) and March 28 to April 25 (post-intervention). Secondary endpoints included the rate of patients receiving empiric antibiotics, hospital length of stay (LOS), 30-day readmissions (for suspected bacterial pneumonia, based on documentation of antibiotic indication), inpatient mortality (all-cause), re-initiation of antibiotics following discontinuation during the same admission (for any indication or specifically for suspected pneumonia based on documentation of antibiotic indication), and rates of *Clostridioides difficile* infections. *C. difficile* infection was defined as a positive *C. difficile* test in conjunction with symptoms of diarrhea requiring treatment.

Categorical data were analyzed with a Fisher’s exact test or a Chi-Square test. Continuous data were analyzed by the Shapiro-Wilk test to determine if the data were normally distributed. Continuous data were analyzed with Student’s t-test for parametric data or a Mann-Whitney U Test for non-parametric data. The significance level for all tests were set at alpha = 0.05. All statistical analyses were performed with STATA®, version 16, College Station, TX.

## Results

A total of 506 inpatients with COVID-19 were screened for inclusion (102 patients in the pre-intervention and 404 patients in the post-intervention). One hundred and fifty-five patients were excluded because they did not receive any antibiotics during the admission, 80 patients were excluded because a source of infection other than pneumonia was identified during the admission, and 24 patients were excluded because antibiotics were initiated greater than 48 h following admission. A total of 246 patients received empiric antibiotics for CABP and were included in the antibiotic duration and clinical outcomes analysis, with 76 patients in the pre-intervention group and 170 in the post-intervention group. Baseline characteristics are shown in Table 1. More patients in the post-intervention group had a fever (55% vs. 25%, *p* = 0.001) and leukocytosis (24% vs. 7%, *p* = 0.002) at the time of antibiotic initiation, while more patients in the pre-intervention group required mechanical ventilation within the first 24 h of admission (11% vs. 24%, *p* = 0.01) and at any point (17% vs. 36%, *p* = 0.02) during the hospital course. Overall, there were a total of 11 (4.5%) non-SARS-CoV-2 respiratory pathogens identified by either RBVP, respiratory culture, or *Streptococcus pneumoniae* urinary antigen tests.

**Table 1:** Baseline characteristics

	Pre-Intervention *N* = 76	Post-Intervention *N* = 170	*p*-value
Age, mean ± standard deviation	58 ± 16.2	61 ± 17	0.20
Male gender	38 (50)	78 (46)	0.65
Race/ethnicity			
Black/African American	71 (93)	151 (88)	0.37
White	3 (4)	10 (6)	0.76
Asian	0 (0)	2 (1)	> 0.99
Other	0 (0)	1 (0.6)	> 0.99
Unknown	0 (0)	4 (2)	0.31
Hispanic/Latino	0 (0)	2 (1)	> 0.99
Hypertension	48 (63)	110 (64)	0.93
Cardiovascular disease	20 (26)	55 (32)	0.44
Diabetes	15 (20)	62 (36)	0.01
Asthma	10 (13)	21 (12)	0.86
Chronic or end stage renal disease	9 (12)	19 (11)	0.87
Immunodeficiency^a^	8 (10)	10 (6)	0.30
COPD	7 (9)	16 (9)	0.97
Obstructive sleep apnea	6 (8)	9 (5)	0.61
HIV	4 (5)	2 (1)	0.07
Bronchiectasis	0 (0)	1 (0.6)	> 0.99
Baseline O2 requirement			
Room air	23 (30)	42 (25)	0.45
Nasal cannula	46 (60)	103 (60)	0.99
High flow nasal cannula	4 (5)	17 (10)	0.32
Non-rebreather	1 (1)	1 (0.6)	0.52
Mechanical ventilation	2 (3)	7 (4)	0.84
ICU admission within first 24 h	21 (28)	51 (30)	0.82
ICU admission at any point	31 (41)	71 (42)	0.99
Mechanical ventilation within first 24 h	18 (24)	18 (11)	0.01
Mechanical ventilation at any point	24 (36)	29 (17)	0.02
Fever at time of antibiotic initiation	19 (25)	93 (55)	< 0.001
Leukocytosis at time of antibiotic initiation	5 (7)	41 (24)	0.002
RBVP obtained	67 (88)	154 (91)	0.72
Positive RBVP	1 (1)	3 (2)	> 0.99
*Legionella* urinary antigen obtained	59 (78)	141 (82)	0.42
Positive Legionella	0 (0)	0 (0)	–
*Streptococcus pneumoniae* urinary antigen obtained	58 (76)	141 (82)	0.30
Positive *Streptococcus pneumoniae*	0 (0)	2 (1.4)	> 0.99
Respiratory cultures obtained	20 (26)	51 (30)	0.70
Positive respiratory culture	1 (5)	4 (8)	> 0.99
Blood cultures obtained	60 (79)	141 (82)	0.57
MRSA swab obtained	42 (55)	125 (74)	0.007
Positive MRSA Swab	1 (2)	6 (8)	0.70
Antibiotics^b^			
Azithromycin	69 (91)	110 (65)	< 0.001
Doxycycline	6 (8)	19 (11)	0.60
Ceftriaxone	64 (84)	112 (66)	0.005
Cefdinir	50 (66)	72 (42)	0.001
Levofloxacin	2 (3)	1 (0.6)	0.22
Cefepime	26 (34)	47 (28)	0.40
Vancomycin	32 (42)	50 (29)	0.07
Amoxicillin-clavulanate or Ampicillin-sulbactam	4 (5)	4 (2)	0.26
Metronidazole	9 (12)	15 (9)	0.60
Other	6 (8)	7 (4)	> 0.99
Antivirals (COVID-19 Directed Therapy)			
HCQ^c^	56 (74)	43 (25)	< 0.001
Tocilizumab	17 (22)	41 (24)	0.90
Remdesivir^d^	24 (32)	48 (28)	0.70
None	11 (14)	67 (39)	< 0.001

^a^ Including transplant patients currently on immunosuppression or patients with malignancy and received chemotherapy or radiation within the past 3 months or Acquired Immunodeficiency Syndrome (AIDS)

^b^Antibiotics initiated empirically for CABP within the first 48 h

^c^ Given alone, or in combination with LPV/r or RBV

^d^ Includes compassionate use or trial Remdesivir

*Abbreviations*: *HCQ* hydroxychloroquine, *LPV/r* lopinavir/ritonavir, RBV: ribavirin

All data are n (%), unless otherwise noted

Following our intervention, 42% of patients (170/404 total patients with COVID-19, post-intervention) received empiric CABP antibiotics compared to 74.5% (76/102 total patients with COVID-19, pre-intervention), *p* < 0.001. Additionally, more patients in the post-intervention group were initiated on antibiotics based on criteria consistent with our guideline (*n* = 52 (68%) versus *n* = 148 (87%), *p* = 0.001). In the post-intervention group, we observed a significant reduction in the number of patients being prescribed azithromycin (91% versus 65%, *p* < 0.001), ceftriaxone (84% versus 66%, *p* = 0.005), and cefdinir (66% versus 42%, *p* = 0.001).

**Fig. 1:**
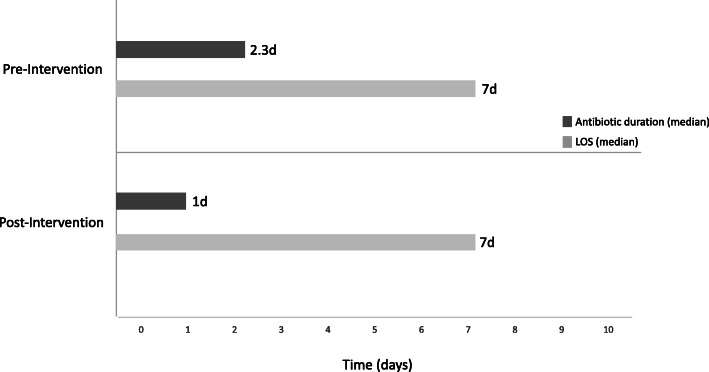
Antibiotic duration and length of stay pre- and post-intervention

The median antibiotic duration of therapy in the post-intervention group was 1.3 days shorter (2.3 versus 1 day, *p* < 0.001) than the pre-intervention group (Fig. 1), and the duration of atypical antibiotic coverage (azithromycin, doxycycline, levofloxacin) was reduced by 2.8 days, (3.8 versus 1 day, p < 0.001). (Table 2) There was no difference between groups in terms of *Clostridioides difficile* infections, the need for antibiotic re-initiation, all-cause readmission rate, mortality rate, or length of stay (Table 2 and Fig. 1). One patient in the pre-intervention group (1.3%) and 3 patients (1.8%) in the post-intervention group were readmitted for suspected bacterial pneumonia (*p* > 0.99). Six (8%) patients in the pre-intervention group and 24 (14%) in the post-intervention group were reinitiated on antibiotics (*p* = 0.24). The reason for reinitiating antibiotics was documented as hospital acquired pneumonia in 2 (2.6%) patients pre-intervention and 15 (8.8%) patients in the post-intervention group (*p* = 0.1). No patients in the pre-intervention group and one (0.6%) in the post-intervention were reinitiated on antibiotics for the indication of CABP (p= > 0.99).

**Table 2:** Antibiotic duration and clinical outcomes

	Pre-Intervention (N = 76)	Post-Intervention (N = 170)	p-value
All antibiotics duration, median days (IQR)	2.3 (1, 3.9)	1 (0.5, 2.1)	< 0.001
Atypical coverage duration, median days (IQR)	3.8 (3, 4.1)	1 (0.4, 1.6)	< 0.001
*Clostridioides difficile* infection	1 (1)	2 (1)	> 0.99
Antibiotics re-initiated			
Any-indication	6 (8)	24 (14)	0.2
Bacterial pneumonia^a^	2 (2.6)	16 (9)	0.07
Readmission within 30 days			
All-cause	5 (7)	23 (13.5)	0.2
Bacterial pneumonia	1 (1.3)	3 (1.8)	> 0.99
Mortality (all-cause)	13 (17)	21 (12)	0.42
Length of stay, median (IQR)	7 (4, 13.2)	7 (4, 12)	0.5

^a^ Two and 15 patients respectively were reinitiated on antibiotics for the indication of hospital acquired pneumonia or ventilator associated pneumonia, 1 patient in the post-intervention group was reinitiated on antibiotics for suspected CABP

## Discussion

Following the implementation of a guideline outlining antibiotic use for bacterial pneumonia among COVID-19 inpatients, we observed a 32.5% absolute reduction in antibiotic prescribing and a 1.3-day shorter duration of therapy. The reduction in duration of therapy was most pronounced with antibiotics targeted at atypical pathogens (e.g. azithromycin, doxycycline, levofloxacin) likely due to guideline recommendations to utilize the RBVP panel and *Legionella* urinary antigen results to aid in de-escalation decisions. Of note, at no point was the use of azithromycin recommended as part of the institution’s COVID-19 treatment guideline (in the absence of possible bacterial pneumonia). As every patient received an ID consultation, we were able to monitor this practice directly. Similar to previous reports [1–4], we observed a high rate of antibiotic prescribing (49%, 246/505) among patients admitted with COVID-19 despite available data suggesting that bacterial co-infection is uncommon among patients with the disease. Our data similarly reflects low rates (4.5%) of co-infection with bacterial pathogens which further supports a need for stewardship interventions to reduce antimicrobial prescribing in this patient population. We found that guideline implementation reinforced by ID COVID-19 Consultation Service recommendations was able to fill this need and increase appropriate antibiotic initiation and de-escalation for CABP in this population.

We observed a higher percentage (non-statistically significant) of patients being re-initiated on antibiotics in the post-intervention group for the indications of hospital-acquired or ventilator-associated pneumonia. Whether the continuation of empiric antibiotics initiated within 48 h of admission for CABP would have prevented the need to reinitiate antibiotics in these patients is unclear, though unlikely as antibiotics initiated for CABP would have likely been narrower in spectrum than what would be necessary to treat nosocomial pathogens.

Other targets for antimicrobial stewardship interventions include duration of therapy, guideline concordant selection of antibiotics, and intravenous to oral conversion of antibiotics [8–19]. Stewardship interventions targeting these aspects of antibiotics for the indication of CABP have been found to be associated with reduced length of stay [12, 18], improved concordance with guideline recommended management (antibiotic selection and duration) [13–16, 19], reduced duration of IV antibiotics [10, 17, 18], and fewer adverse drug reactions [12]. While the benefits of stewardship interventions for patients with CABP in general has been shown in these previous studies, this is the first study to evaluate the impact of ASP on antibiotic use for CABP among patients with COVID-19.

To our knowledge, this is the first report of an antimicrobial stewardship intervention to reduce the prescribing of empiric antibiotics for CABP in COVID-19 patients. Reductions in antibiotic use have important implications and can potentially reduce antimicrobial resistance and antibiotic-related toxicities [2]. Several previous studies have evaluated the impact of stewardship interventions on CABP therapy among the general population. Similar to our findings, most of these studies found no difference in clinical outcomes, suggesting a lack of harm with reduced antibiotic use [8–10]. Furthermore, two previous studies have identified a mortality benefit with antibiotic de-escalation in the setting of CABP (15.1% vs. 25%, *p* = 0.04 and 1.8% vs. 5.5%, p = 0.04), and one found a significantly reduced length of stay (5 vs. 9 days, *p* < 0.001) [10, 11]. Additional findings that support the safety of reduced antibiotic prescribing in our study include similar rates of antibiotic re-initiation and readmission between groups.

There are a few pertinent limitations to outline. Given the quasi-experimental study design, there are several confounders that may have contributed to the study results. First, the higher rate of mechanical ventilation in the pre-intervention group suggests the disease severity at baseline may have differed between groups. However, this difference may be attributed to changes in critical care practice in utilization of non-invasive ventilatory interventions such as proning, high flow nasal cannula, and helmet ventilation [20]. Additionally, more patients in the post-intervention group had fever and leukocytosis, which also speaks to impact of the intervention in terms of facilitating appropriate initiation of empiric antibiotics based on the presence of fever and/or leukocytosis. Second, after several weeks of managing COVID-19 inpatients, there was likely improved clinician comfort with COVID-19 management as well as more data suggesting low concern for bacterial co-infection during the post-intervention period. Third, changes in SARS-CoV-2 testing may have resulted in a reduced turnaround time in the post-intervention period. Although the timeliness of the SARS-CoV-2 test result may not have had a direct impact on prescribing empiric antibiotics, this may have contributed to a longer duration of antibiotics in the pre-intervention period. Fourth, this study was underpowered and not designed to investigate clinical outcomes such as adverse drug effects, mortality, and length of stay. Lastly, while developing a guideline is a simple intervention, reinforcement of the guideline on daily ID consult rounds may be difficult to implement. The results of our study may not be generalizable to other medical centers that may not have the capacity or resources available to provide daily review. Though we did not study this, prospective audit with feedback and intervention and/or ASP rounds may be a suitable alternative since ID consults did not involve direct patient examination.

## Conclusions

A targeted clinical guideline implemented by an ASP/ID COVID-19 consult service was an effective tool to reduce inappropriate prescribing of antibiotics for CABP in patients with COVID-19 pneumonia. Additional studies are needed to further explore the potential clinical impact of stewardship interventions targeting prescribing of antibiotics for CABP among patients with COVID-19.

## Supplementary Information

**Additional file 1: Supplement Figure 1**: COVID-19, CABP Antibiotic Initiation and Discontinuation Guideline.

## Data Availability

The de-identified datasets used and/or analyzed during the current study are available from the corresponding author on reasonable request.
