# Supplementary material for: Reducing the use of empiric antibiotic therapy in COVID-19 on hospital admission
Source: BMC Infect Dis. 2021 Jun 2;21:516. doi: 10.1186/s12879-021-06219-z (PMC8170434; doi:10.1186/s12879-021-06219-z)
Supplement: Supplementary file 1 — Additional file 1: Supplement Figure 1: COVID-19, CABP Antibiotic Initiation and Discontinuation Guideline. [file 12879_2021_6219_MOESM1_ESM.docx]

Supplementary Material

Supplement Figure 1: COVID-19, CAP Antibiotic Initiation and Discontinuation Guideline
